# In and out among the Hospitals

**Published:** 1888-04-21

**Authors:** 


### In and Out Among the Hospitals.

By a Secretary of Ten Years' Standing.

Copies of Annual Reports and Rules, Accounts of Meetings, Changes in the Staff, &c., should be addressed The Editor, The Lodge, Porchester Square, W.

### THE RADCLIFFE INFIRMARY, OXFORD.

The report of the Radcliffe Infirmary, Oxford, gives a flourishing account of last year's work. The receipts from almost all sources have been above the average; the total of income being £7,888 6s. 4d., and the total of expenditure £5,323 17s. 1d. Though the committee have been put to considerable expense for extraordinary work in connection with certain structural alterations and improvements in the water supply, the floating debt which has so long hampered this Institution has been virtually extinguished. The fever wards have been more than ordinarily full in consequence of a short outburst of scarlet fever which occurred during the year. These patients usually pay for their treatment, and this has aided the Charity. A special Jubilee Fund was raised in the County and City, the aggregate amount collected being £1,097. With this the governors have determined to carry out an extension of the female block, in accordance with plans that have already been made and approved. The estimated cost of this work is almost exactly covered by the Jubilee Fund, and it is proposed, when the building is completed, to call the block "The Queen's Block." There is a general tendency to improve at the Radcliffe Infirmary, and already special arrangements have been made for the treatment ef diseases of the eyes and teeth; besides that, it is under contemplation to establish a special department for the treatment of diseases of women. The daily average number of beds occupied was 102, and the cost per occupied bed, worked out by the expenditure of the year after deducting 1*s.* for each out-patient, was £51 18*s.* This, it is stated, is a reduction on last year. The report is admirably got up, the accounts are clearly and accurately set forth, and the information given is all that could be desired by the most critical governor.

### NORFOLK AND NORWICH INFIRMARY.

Every charge against this Hospital up to the 31st of December last has been paid off, leaving a balance of £1,750 4*s.* 1*d.*, which has been set aside for investment. The income for the year was £8,418 14*s.* 6*d.*, the expenditure £7,276 1*s.* 2*d.*; a very satisfactory result, and it is to be wished that the financial status of every county hospital was the same. The Norfolk and Norwich Hospital is an ably administered charity, because every member of the board of management takes an active interest in its welfare. The buildings are spacious and well-appointed, being surrounded by gardens for the recreation of patients. The Rev. J. Callis having resigned the post of Chaplain, the Bishop and Dean of Norwich, in whom the right is vested, have appointed the Rev. G. Willoughby Barrett to fill the vacancy thus created. Mr. W. A. Earnshaw, the late Secretary, has been succeeded by Mr. A. E. Boyce, of the General Hospital, Birmingham. The daily average number of patients was 136½, being 13 more than in the previous year, and the average cost per bed, calculated upon the total expenditure of the year, was £53 6*s.* 1*d.*, or deducting out-patients at 1*s.* per head, £38 15*s.* 1*d.* The total number of in-patients was 1,352, as against 1,124 in 1886.

### THE KILMARNOCK INFIRMARY.

The report of this infirmary has just reached us. The accounts are made up from the 11th of November, 1886, to the 11th of November, 1887 (why a year and a day is not explained). In common with many other of the smaller Provincial Hospitals, the Governors have had again to face the painful experience of an increase of expenditure and inadequate receipts. The expenditure has been £1,650 18*s.* 2*d.*, and the receipts £1,774 12*s.* 6*d.*; but there are some hundreds still due to the bank. The total number of patients was 529, and the daily average 42. This number gives the cost per bed occupied at £39 6*s.* The number of in-patients exceeds that of any former year, and the difficulty of financing the Infirmary that thus confronts the Directorate is very serious. It does not appear that any out-patients are treated—at any rate, no such record is found in the printed tables. The bright side of the report is that of the physicians and surgeons, and is well worthy of careful perusal. The death-rate is unusually low, and the results of operations have been satisfactory. Dr. Macfarlane resigned his appointment as one of the physicians, as he intends practising in London, and the vacancy thus caused in the staff has been filled up by the election of Dr. W. A. Macleod. A feature of this Infirmary is that there is an ambulance waggon always in readiness. This has been presented by Mrs. and the Misses Finnie, of Springhill, and it has been found of very great service. The use of the waggon is not restricted to Infirmary patients only, but is available for the use of the district generally.

